# Detection of Integrase Gene in *E. coli* Isolated from Pigs at Different Stages of Production System

**DOI:** 10.1155/2014/489569

**Published:** 2014-03-10

**Authors:** Eulalia de la Torre, Rocío Colello, Nora Lía Padola, Analía Etcheverría, Edgardo Rodríguez, Fabián Amanto, María Ofelia Tapia, Alejandro Luis Soraci

**Affiliations:** ^1^Área de Toxicología, Departamento de Fisiopatología, Centro de Investigación Veterinaria de Tandil-Consejo Nacional de Investigaciones Científicas y Técnicas-Comisión de Investigaciones Científicas Provincia de Buenos Aires (CIVETAN-CONICET-CICPBA), Facultad de Ciencias Veterinarias, Universidad Nacional del Centro de la Provincia de Buenos Aires (UNICEN), Campus Universitario, Paraje Arroyo Seco s/n, Tandil, Argentina; ^2^Área de Inmunoquímica y Biotecnología, Departamento de Sanidad Animal y Medicina Preventiva (SAMP), CIVETAN-CONICET-CICPBA, Facultad de Ciencias Veterinarias, UNICEN, Campus Universitario, Paraje Arroyo Seco s/n, Tandil, Argentina; ^3^Área de Bioestadística, Departamento de SAMP, CIVETAN-CONICET-CICPBA, Facultad de Ciencias Veterinarias, UNICEN, Campus Universitario, Paraje Arroyo Seco s/n, Tandil, Argentina; ^4^Área de Producción Porcina, Departamento de Producción Animal, Facultad de Ciencias Veterinarias, UNICEN, Campus Universitario, Paraje Arroyo Seco s/n, Tandil, Argentina

## Abstract

Integrons are one of the genetic elements involved in the acquisition of antibiotic resistance. The aim of the present research is to investigate the presence of integrons in commensal *Escherichia coli* (*E. coli*) strains, isolated from pigs at different stages of production system and from the environment in an Argentinian farm. Five sows postpartum and five randomly chosen piglets from each litter were sampled by rectal swabs. They were sampled again at day 21 and at day 70. Environmental samples from the farm were also obtained. *E. coli* containing any integron class or combination of both integrons was detected by polymerase chain reaction in 100% of sows and in piglets at different stages of production: farrowing pen stage 68.1%;, weaning 60%, and growing/finishing 85.8%, showing an increase along the production system. From environmental samples 78.4% of *E. coli* containing any integron class was detected. We conclude that animals and farm environment can act as reservoirs for potential spread of resistant bacteria by means of mobile genetic elements as integrons, which has a major impact on production of food animals and that can reach man through the food chain, constituting a problem for public health.

## 1. Introduction

During the last years, the use of antimicrobial agents has markedly increased, both in human and veterinary medicine [1]. The main problem with the indiscriminately used antimicrobial agents to treat infections is the development of resistant strains of pathogenic bacteria. This issue is of major concern to human and animal health, mainly because it leads to a greater risk of therapeutic failure of standard infection management [2–4]. Several antimicrobials are used worldwide as growth promoters, and as metaphylactic, prophylactic, and therapeutic agents. There is growing evidence that antimicrobials used in animal feed exert a strong selective pressure that stimulates the development of antibiotic resistance in enteric bacteria [1, 5, 6]. The presence of antimicrobial agents allows the selection of resistance genes in nonpathogenic bacteria, which, in time, can be horizontally transmitted to different species of pathogenic or zoonotic microorganisms [7–9]. Horizontal gene transfer seems to be the main cause of the rapid proliferation of antibiotic-resistance genes across a wide diversity of bacteria [10].

Beyond the horizontal gene transfer, the loss and acquisition of functional modules are important in the process of rapid bacterial development of resistance [11, 12]. Integrons are one of the genetic elements involved in the acquisition of antibiotic resistance; they are capable of capturing, integrating, and mobilizing antibiotic resistant gene cassette [13]. Integrons lack the specific mobilization machinery, but they are associated with insertion sequences, transposons, and/or plasmids. The functional arrangement of integrons is composed of the* intI* gene, which encodes an integrase that catalyzes the specific excision and integration of gene cassettes, a specific recombination site* attI*, and a promoter [14]. There are two main groups of integrons: group I, also called mobile integrons, related to antibiotic resistance cassettes, and group II or superintegrons, which, unlike the first ones, are present at a chromosomal level, and they are not related to antibiotic resistance with a few exceptions. Group I, the one we are focused on, is in turn divided into five classes: integrons class 1, class 2, class 3, class 4, and class 5 based on the sequence of the encoded integrases. Although only the first three have been historically involved in the spread of multiresistance phenotypes, all five classes have been associated with antibiotic-resistance determinants [15]. It is known that the presence of integrase is potentially indicative of strains capable of recruiting antibiotic resistance genes [16].

The animal gastrointestinal tract is the main reservoir and spread site for bacteria, but also a special environment for genetic information exchange [8, 17–20]. Therefore, surveillance of commensal microflora, such as* Escherichia coli* (*E. coli*) strains, is a good indicator of resistance patterns within a population, as they are frequently found and readily acquire resistance [21].

Considering what has been formerly explained, the aim of the present research is to investigate the presence and frequency of integrons in commensal* E. coli* strains, isolated from pigs at different stages of production system and from the environment in an Argentinian farm.

## 2. Materials and Methods

### 2.1. Management of Farm and Animals

Samples were taken from a commercial farm in Buenos Aires province, Argentina, between March and June 2012. The farm is intensively organized in total confinement, with 400 females in production. The main facilities are gestation, farrowing, weaning, and growing/finishing (fattening), which are geographically separated from each other within the same farm. Gestation facilities consist in rectangular sheds with two rows of cages to house each sow individually, in order to have a strict control of the sow from the time of insemination to its transfer to the farrowing area (approximately 110 days). Farrowing installation comprises a set of rooms with pit. Each room consists of a set of iron farrowing crates where sows are housed four days before birth and remains with the litter until weaning. When the litter reaches 70 days and a weight of 35 kg is transferred to fattening or termination area, this facility consists on a large rectangular enclosure capable of holding 0.7 m^2^/animal. Each enclosure is divided into rooms and each room consists in a variable number of pens depending on the size of the group. The partitions between pens are made of concrete. The usual group size varies between 10 and 30 pigs. Pigs and employees move from one building to another by means of corridors that are isolated from external traffic.

For the present study five sows of the same genetic line were used. Selection was based on the following productive criteria: 2.4 births per sow per year, >22 pigs weaned per sow per year, and >27 pigs born alive per sow per year.

Antibiotics use is restricted as claimed by producer at detailed interviews. Subtherapeutic feed-based antibiotics were not used, or only subtherapeutic concentrations of tetracyclines were used for short period during disease outbreaks. On the other hand, injectable antibiotics (fosfomycin, tiamulin, and amoxicillin) were not used except for brief periods to control atypical disease outbreaks (less than 15 days per year).

### 2.2. Sample Collection

Five sows were randomly selected and fecal material was collected via rectal swab at 3 h postpartum. Swabs were also obtained from five randomly chosen piglets from each test sow on the same day. The piglets selected from each litter were sampled again at day 21 and at day 70. These ages corresponded to phases in which the pigs were housed in the farrowing crate (F), weaning (G), and growing/finishing units (G/F), respectively. The swabs were transported into Stuart medium. Water samples (30 mL) were collected from the farrowing pen, weaning and growing/finishing pit, treatment chamber, and waste treatment lagoon. The water sampling was performed in parallel with the samples of pigs, attending to routine cleaning and emptying of the pit of the farm, and according to the guidelines established in the Manual for Basic Analysis of the Drinking Water Quality of the World Health Organization (2004). All samples were transported refrigerated and processed immediately at laboratory. In Table 1, the total number of animals tested and samples obtained are detailed.

### 2.3. Isolation of* E. coli* Strains

Faecal samples and 10 mL of water were grown in 20 and 100 mL of LB broth, respectively, for 24 h at 37°C, with shaking. Then 10 *μ*L was plated on MacConkey agar and incubated for 18 h at 37°C. From each plate, suspected* E. coli* isolates (5–7 per plate) were randomly selected and were cultured in 0.8 mL of LB for 24 h at 37°C, with shaking for DNA extraction. In Table 2, the number of isolates obtained from the animals (a) and the environment (b) are shown.

The DNA template was isolated by incubating 10 *μ*L of bacterial culture in 500 *μ*L of sterile bidistilled H_2_O at 100°C for 10 min.

### 2.4. Identification of* E. coli* Strains by PCR

*E. coli* gene universal stress protein (uspA) was used to confirm* E. coli *[22]. Virulence genes* stx1*,* stx2*,* stx2e, eae*,* lt*,* sta*, and* stb* characteristic of verotoxigenic* E. coli* (VTEC), enteropathogenic* E. coli* (EPEC), and enterotoxigenic* E. coli* (ETEC), respectively, were detected by PCR. Only negative strains for these genes have been considered as commensal and have been included in this study [23].

### 2.5. Integrons Detection by PCR

Genes encoding integrase 1 (*Int1*) and integrase 2 (*Int2*) were detected to identify the presence of integrons class 1 or class 2, respectively, according to Orman et al. [24] with modifications. Briefly the thermal cycling conditions modified were preincubation (95°C for 10 min), followed by 30 cycles of denaturation (94°C for 1 min), annealing temperature 57°C and 55°C for 1 min (*Int1* and* Int 2*, resp.), and extension (72°C for 10 min).

### 2.6. Statistical Analysis

The variable presence or absence of integrons was analyzed using the PROC GENMOD procedure of SAS 9.2 (SAS Institute Inc., Cary, NC, USA). The odds ratio (OR) was estimated by contrasts (CLI: confidence limits interval).

## 3. Results

### 3.1. Presence of Integrons in* E. coli* Recovered from Sows

**Figure 1:**
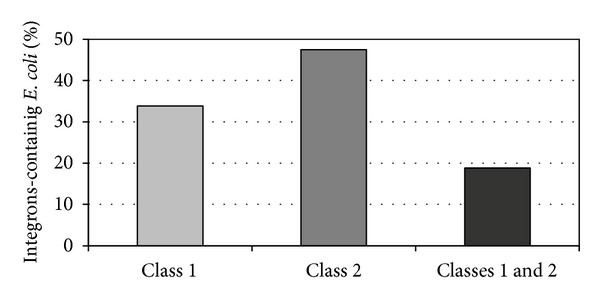
Percentages of* E. coli* integron class 1 and/or class 2 positive isolates in sows.

*E. coli* containing any integron class or combination of both integrons was detected by PCR in 5 sows tested (100%, Table 1). From 80* E. coli* isolates, 33.8% were integron class 1 positive (ECi1+), 47.5% were integron class 2 positive (ECi2+), while 18.8% were positive for both integrons (ECi1+2+) (Figure 1).

### 3.2. Presence of Integrons in* E. coli* Recovered from Piglets at Different Stages of Production

**Figure 2:**
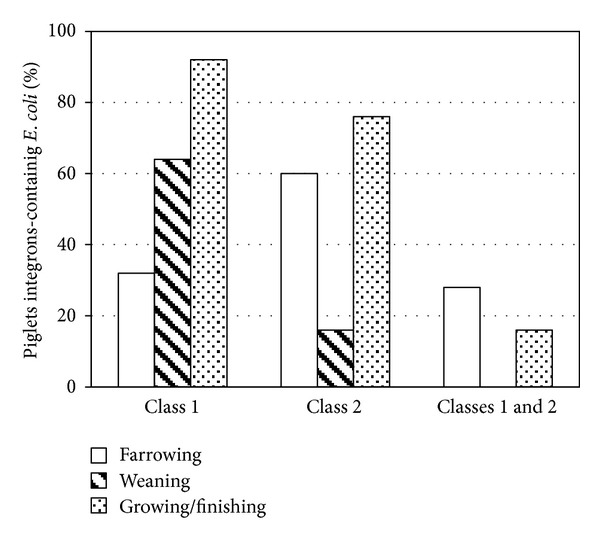
Percentages of integron positive piglets at different stages of production.

During the growing/finishing stage, the highest percentage of piglets containing ECi1+ and ECi2+ (91.7% and 75%, resp.) was found (Table 2). We observed a significant increase in the amount of piglets harboring ECi1+ as they moved to different stages of production (*P* < 0.05), while there was a significant decrease of piglets harboring ECi2+ during weaning stage (*P* < 0.004) (Figure 2).

**Figure 3:**
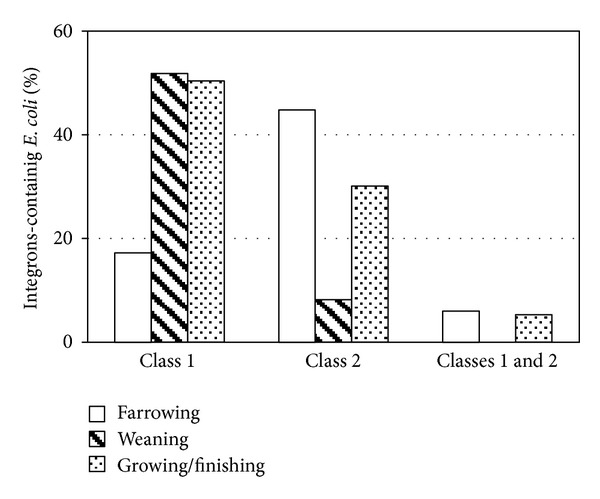
Percentages of* E. coli* integron positive strain obtained from piglets isolates at different stages of production.

In piglets, 314* E. coli* isolates were obtained.* E. coli* isolated from piglets showed the following percentages of any integron class during the different stages of production: farrowing pen stage 68.1%, weaning 60%, and growing/finishing 85.8% showing an increase along the production system (Table 2). Furthermore,* E. coli* ECi1+ significantly increased from the first stages of production, being predominant at growing/finishing (*P* < 0.0001). On the contrary, ECi2+ was more frequent during the farrowing pen (44.8%) decreasing significantly during weaning (8.2%, *P* < 0.0001). Therefore, we observed that during weaning or growing/finishing there are about 5 times more chances of finding strains of ECi1+ (OR_W_ = 5.03 CLI95% = 2.65–9.55 and OR_G/F_ = 4.89 CLI95% = 2.66–8.96) and less chance of finding Eci2+, than at farrowing pen stage (OR_W_ = 0.10 CLI95%: 0.05–0.26 and OR_G/F_ = 0.53 CLI95%: 0.31–0.91). Strains harboring ECi1+2+ were detected at farrowing pen (6%) and at growing/finishing (5.3%) (Figure 3). Notably, all sows had at least one piglet in which ECi+ was isolated.

### 3.3. Detection of Integrons in the Environment of the Farm

**Figure 4:**
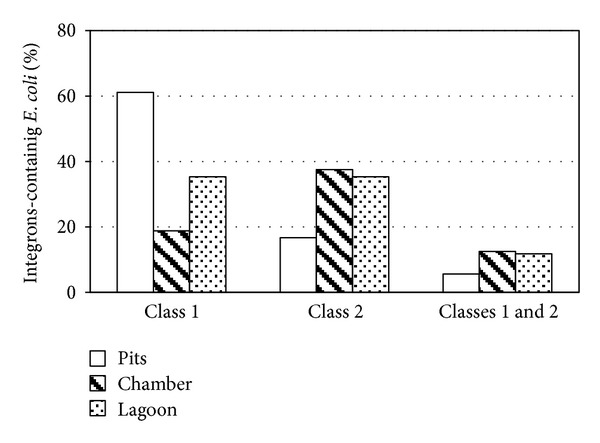
Percentages of* E. coli* integron positive isolates in pits production rooms for pigs, treatment chamber, and waste treatment lagoon in pig farm.

Since commensals* E. coli* isolates obtained from sows and piglets contained integrons, the presence of this genetic element in water samples from different places of the pig farm was investigated. Fifty-one isolates were obtained from different environmental samples. Seventy- eight percent of* E. coli* containing any integron class was detected. We observed that in environmental samples there was a higher percentage of ECi1+ compared to ECi2+ (39.2% and 29.4%, resp.), while a low percentage was positive for both integrons (9.8%). In the production pits, the presence of ECi1+ (61.1%) was more frequent compared to the ECi2+ (16.7%) or the combination of both integrons (5.6%). The highest percentage of ECi2+ and ECi1+2+ was recorded in water samples from treatment chamber (37.5% and 12.5%, resp.) and lagoon (35.3% and 11.8%, resp.) (Figure 4).

## 4. Discussion

Antibiotics are commonly used to control livestock diseases, for prophylaxis and for growth promotion. These practices can select antibiotic-resistant organisms in the gastrointestinal tract of animals, especially when used in subtherapeutic levels [25]. The antibiotic resistance among bacteria can be transmitted by a variety of mobile genetic elements such as plasmids, transposons, and integrons [8, 9]. We have demonstrated that commensal* E. coli* isolated from sows from a farm in Argentina harbored the genes for integrases 1 and/or 2 suggesting that there are strains capable of recruiting antibiotic resistance genes [16]. The emergence of antibiotic-resistant commensal bacteria has been studied extensively in food animals worldwide. The high prevalence of commensal* E. coli* antibiotic-resistant strains in pigs is alarming [26–28], due to the potential to transfer resistance determinants to other bacteria, including human pathogens. Mathew et al. [29] demonstrated that identical integrons were found in two different species of bacteria,* E. coli* and* Salmonella*, from one pig farm, suggesting that horizontal transfer has occurred between these two organisms. Additionally, several authors have shown that a high percentage of* E. coli* strains from food animals such as cattle, pigs, and poultry contain integrons [19, 30–32]. The fact that integrons are present in conjugative plasmids and transposons, which facilitates conjugation, explained its wide distribution [33].

We also demonstrated the presence of integrons in commensal* E. coli* strains in piglets younger than 12 h of age suggesting the importance of the transmission of resistant strains in the litter at birth. Moreover, we noted that the percentage of strains of* E. coli* positive for some type of integrons increased during the different stages of production system. Marchant and Moreno [34] observed a high percentage of* E. coli* isolates containing integron positive within the first 8 weeks of life, which decreases to 21 weeks. Andraud et al. [35] observed that the transmission of resistant bacterial strains between individuals is a key enabling persistence in pig farms. Furthermore, most of studies do not consider the age of the animals. Our data indicate that the age is an important factor influencing the presence of some class of integron in pigs. It must be kept in mind that at weaning, the liquid or/and semiliquid diet becomes dry food. So, the piglets are exposed to antibiotics used in the food, in some countries, as growth promotion [36–38]. Among the different classes of integrons, the most frequently described and related to antibiotic resistance in Gram-negative bacteria is integron class 1, which to date has over 100 different cassette resistance arrangements informed [16, 39–41]. We observed that the integron class 1 predominated in the later stages of pig production, while class 2 was more frequent in sows and farrowing crate stage, possibly reflecting the mother's bacterial load which changes over the weeks. Literak et al. [42] observed that sows contained higher percentage of* E. coli* integron class 2 positive compared to class 1 in farm where antibiotics are commonly used. Marchant and Moreno [34] demonstrated that unweaned piglets had a higher percentage of* E. coli* integron class 2 versus class 1. This is reversed in growers piglets (about 56 days), consistent with our results, piglets of 70 days. Machado et al. [43] found a higher percentage of integron class 1 compared with class 2 in* E. coli* isolates from healthy adult pigs. Contrary to our results, Campbell et al. [44] found significant differences between groups of swine production. The group of older animals had a lower percentage of bacteria positive for integron class 1 compared to the group of younger animals. Other authors have also shown a widespread of integron class 1 in Enterobacteriaceae family members from livestock, pets, and exotic animals [45]. Therefore, the development, persistence, and spread of resistant bacteria are of increasing concern, because they could compromise infection control by reducing treatment options for infected animals. This may lead to an overall increase in disease transmission, morbidity, mortality, and sometimes economic losses to the animal production industry [46, 47]. As we mentioned, integrons are among genetic elements that have contributed to the development of multidrug resistance in Gram-negative bacteria. We have observed a high percentage of multidrug antibiotic resistance strains from pigs. In antibiotic susceptibility tests, the highest percentage of resistance was observed for tetracycline, tiamulin and enrofloxacin. Resistance to more than two antibiotics increases during the latter stages of production (data not shown).

The persistence of resistant strains among individuals in a farm may involve dissemination within the environment. Well and pond systems are typically used to manage the manure produced on a farm. This causes great concern that antibiotic-resistant bacteria could easily migrate into soil, groundwater, and surface water through seepage from manure lagoons and from the application of manure [48, 49]. We have shown that, in the farrowing pen, weaning and growing/finishing piglets pits, treatment chamber, and waste treatment lagoon, there is a high percentage of ECi+, being the percentage of Eci1+ more higher than Eci2+ and Eci1+2+. Byrne-Bailey et al. [50] demonstrated a higher prevalence of integron class 1 positive bacteria in soils treated with pig slurry versus integron class 2, which remains high after 10 months. However, we observed differences in the integron class depending on the place where the sample was taken. In pits production rooms for pigs, integron class 1 predominates, while integron class 2 predominates in treatment chamber. In water samples from the treatment lagoon, where the effluent water and all residual contaminants are disposed, the percentage of integron class 1 and class 2 positive strains were the same. Other authors have found a prevalence of integrons class 1 and 2 in slaughterhouse wastewater treatment plants of 30.7% and 4.5%, respectively [51]. These variations in the presence or frequency of integrons class, both in the environment and in animals, may be due to the different policies of use and/or abuse of antibiotics used at different production farms. Literak et al. [42] investigated two swine production farms with different policies on the use of antibiotics. They found a higher prevalence of integrons in isolates of* E. coli* from pigs from a farm where the use of several antibiotics was common, compared to a farm that manages antibiotics only exceptionally. This suggests the important effect of selection pressure exerted by antibiotics. We must clarify that the diversity of isolates has not been investigated, so it is possible that some studied isolates belong to the same clone.

Therefore, we conclude that animals and farm environment can act as reservoirs for potential spread of resistant bacteria by means of mobile genetic elements as integrons, which are involved in the antibiotic resistance and which has a major impact on production of food animals and that can reach man through the food chain, constituting a problem for public health.

## Figures and Tables

**Table 1:** Details of the total number of animals tested and samples obtained.

	Number of animals	Number of animals containing *E. coli* integron positive	Number of animals containing *E. coli* integron class 1 positive	Number of animals containing *E. coli* integron class 2 positive	Number of animals containing *E. coli* integron classes 1 and 2 positive
Sows	5 (15*)	5	5	5	5
Piglets F	25	18	8	15	7
Piglets W	25	15	13	3	0
Piglets G/F	25	24	22	18	16

*Each sow was sampled 3 times in each sampling occasion of different stage of pig production. Piglets F (Farrowing), W (Weaning) y G/F (Growing/Finishing).

**Table:** (a)

	Commensal *E. coli* isolates	*E. coli* isolates integron class 1 positive	*E. coli* isolates integron class 2 positive	*E. coli* isolates integron classes 1 and 2 positive
Sows	80	27	38	15
Piglets F	116	20	52	7
Piglets W	85	44	7	0
Piglets G/F	113	57	34	6

**Table:** (b)

	Number of samples	Commensal *E. coli* isolates	*E. coli* isolates integron class 1 positive	*E. coli* isolates integron class 1 positive	*E. coli* isolates integron classes 1 and 2 positive
Pits	3	18	11	3	1
Chamber	3	16	3	6	2
Lagoon	3	17	6	6	2

Piglets F (Farrowing), W (Weaning) y G/F (Growing/Finishing).

## References

[1] Angulo FJ, Nargund VN, Chiller TC (2004). Evidence of an association between use of anti-microbial agents in food animals and anti-microbial resistance among bacteria isolated from humans and the human health consequences of such resistance. *Journal of Veterinary Medicine B*.

[2] Teale CJ (2002). Antimicrobial resistance and the food chain. *Journal of Applied Microbiology*.

[3] Snary EL, Kelly LA, Davison HC, Teale CJ, Wooldridge M (2004). Antimicrobial resistance: a microbial risk assessment perspective. *Journal of Antimicrobial Chemotherapy*.

[4] White DJ, McDermott PF (2001). Emergence and transfer of antibacterial resistance. *Journal of Dairy Science*.

[5] Barbosa TM, Levy SB (2000). The impact of antibiotic use on resistance development and persistence. *Drug Resistance Updates*.

[6] Jensen VF, Jakobsen L, Emborg H-D, Seyfarth AM, Hammerum AM (2006). Correlation between apramycin and gentamicin use in pigs and an increasing reservoir of gentamicin-resistant *Escherichia coli*. *Journal of Antimicrobial Chemotherapy*.

[7] Stobberingh EE, van den Bogaard AE (2000). Spread of antibiotic resistance from food animals to man. *Acta veterinaria Scandinavica. Supplementum*.

[8] Blake DP, Hillman K, Fenlon DR, Low JC (2003). Transfer of antibiotic resistance between commensal and pathogenic members of the Enterobacteriaceae under ileal conditions. *Journal of Applied Microbiology*.

[9] Zhang XY, Ding LJ, Yue J (2009). Occurrence and characteristics of class 1 and class 2 integrons in resistant *Escherichia coli* isolates from animals and farm workers in Northeastern China. *Microbial Drug Resistance*.

[10] Aminov RI (2011). Horizontal gene exchange in environmental microbiota. *Frontiers in Microbiology*.

[11] Wozniak RAF, Waldor MK (2010). Integrative and conjugative elements: mosaic mobile genetic elements enabling dynamic lateral gene flow. *Nature Reviews Microbiology*.

[12] Stalder T, Barraud O, Casellas M, Dagot C, Ploy MC (2012). Integron involvement in environmental spread of antibiotic resistance. *Frontiers in Microbiology*.

[13] Turton JF, Kaufmann ME, Glover J (2005). Detection and typing of integrons in epidemic strains of *Acinetobacter baumannii* found in the United Kingdom. *Journal of Clinical Microbiology*.

[14] Boucher Y, Labbate M, Koenig JE, Stokes HW (2007). Integrons: mobilizable platforms that promote genetic diversity in bacteria. *Trends in Microbiology*.

[15] Cambray G, Guerout A-M, Mazel D (2010). Integrons. *Annual Review of Genetics*.

[16] Di Conza JA, Gutkind GO (2010). Integrons: gene collectors. *Revista Argentina de Microbiologia*.

[17] Nikolich MP, Hong G, Shoemaker NB, Salyers AA (1994). Evidence for natural horizontal transfer of tetQ between bacteria that normally colonize humans and bacteria that normally colonize livestock. *Applied and Environmental Microbiology*.

[18] Knezevic P, Petrovic O (2008). Antibiotic resistance of commensal *Escherichia coli* of food-producing animals from three Vojvodinian farms, Serbia. *International Journal of Antimicrobial Agents*.

[19] Lapierre L, Cornejo J, Borie C, Toro C, San Martín B (2008). Genetic characterization of antibiotic resistance genes linked to class 1 and class 2 integrons in commensal strains of *Escherichia coli* isolated from poultry and swine. *Microbial Drug Resistance*.

[20] Hoa NT, Chieu TTB, Nga TTT (2011). Slaughterhouse pigs are a major reservoir of *Streptococcus suis* serotype 2 capable of causing human infection in Southern Vietnam. *PLoS ONE*.

[21] Caprioli A, Busani L, Martel JL, Helmuth R (2000). Monitoring of antibiotic resistance in bacteria of animal origin: epidemiological and microbiological methodologies. *International Journal of Antimicrobial Agents*.

[22] Chen J, Griffiths MW (1998). PCR differentiation of *Escherichia coli* from other Gram-negative bacteria using primers derived from the nucleotide sequences flanking the gene encoding the universal stress protein. *Letters in Applied Microbiology*.

[23] Parma AE, Sanz ME, Viñas MR (2000). Toxigenic *Escherichia coli* isolated from pigs in Argentina. *Veterinary Microbiology*.

[24] Orman BE, Piñeiro SA, Arduino S (2002). Evolution of multiresistance in nontyphoid *Salmonella* serovars from 1984 to 1998 in Argentina. *Antimicrobial Agents and Chemotherapy*.

[25] Wegener HC (2003). Antibiotics in animal feed and their role in resistance development. *Current Opinion in Microbiology*.

[26] Enne VI, Cassar C, Sprigings K, Woodward MJ, Bennett PM (2008). A high prevalence of antimicrobial resistant *Escherichia coli* isolated from pigs and a low prevalence of antimicrobial resistant *E. coli* from cattle and sheep in Great Britain at slaughter. *FEMS Microbiology Letters*.

[27] Harada K, Asai T, Kojima A, Sameshima T, Takahashi T (2007). Contribution of multi-antimicrobial resistance to the population of antimicrobial resistant *Escherichia coli* isolated from apparently healthy pigs in Japan. *Microbiology and Immunology*.

[28] Phongpaichit S, Liamthong S, Mathew AG, Chethanond U (2007). Prevalence of class 1 integrons in commensal *Escherichia coli* from pigs and pig farmers in Thailand. *Journal of Food Protection*.

[29] Mathew AG, Liamthong S, Lin J, Hong Y (2009). Evidence of class 1 integron transfer between *Escherichia coli* and *Salmonella* spp. on livestock farms. *Foodborne Pathogens and Disease*.

[30] Guerra B, Junker E, Schroeter A, Malorny B, Lehmann S, Helmuth R (2003). Phenotypic and genotypic characterization of antimicrobial resistance in German *Escherichia coli* isolates from cattle, swine and poultry. *Journal of Antimicrobial Chemotherapy*.

[31] Hsu S-C, Chiu T-H, Pang J-C, Hsuan-Yuan C-H, Chang G-N, Tsen H-Y (2006). Characterisation of antimicrobial resistance patterns and class 1 integrons among *Escherichia coli* and *Salmonella enterica* serovar Choleraesuis strains isolated from humans and swine in Taiwan. *International journal of antimicrobial agents*.

[32] Karczmarczyk M, Abbott Y, Walsh C, Leonard N, Fanning S (2011). Characterization of multidrug-resistant *Escherichia coli* isolates from animals presenting at a University Veterinary Hospital. *Applied and Environmental Microbiology*.

[33] Fluit AC, Schmitz F-J (2004). Resistance integrons and super-integrons. *Clinical Microbiology and Infection*.

[34] Marchant M, Moreno MA (2013). Dynamics and diversity of *Escherichia coli* in animals and system management of the manure on a commercial farrow-to-finish pig farm. *Applied and Environmental Microbiology*.

[35] Andraud M, Rose N, Laurentie M (2011). Estimation of transmission parameters of a fluoroquinolone-resistant *Escherichia coli* strain between pigs in experimental conditions. *Veterinary Research*.

[36] Thomke S, Elwinger K (1998). Growth promotants in feeding pigs and poultry. I. Growth and feed efficiency responses to antibiotic growth promotants. *Animal Research*.

[37] Thomke S, Elwinger K (1998). Growth promotants in feeding pigs and poultry. II. Mode of action of antibiotic growth promotants. *Animal Research*.

[38] Weber TE, Schinckel AP, Houseknecht KL, Richert BT (2001). Evaluation of conjugated linoleic acid and dietary antibiotics as growth promotants in weanling pigs. *Journal of Animal Science*.

[39] Mazel D (2006). Integrons: agents of bacterial evolution. *Nature Reviews Microbiology*.

[40] Mosquito S, Ruiz J, Bauer JL, Ochoa TJ (2011). Molecular mechanisms of antibiotic resistance in *Escherichia coli* associated diarrhea. *Revista Peruana de Medicina Experimental y Salud Pública*.

[41] Povilonis J, Šeputiene V, Ružauskas M (2010). Transferable class 1 and 2 integrons in *Escherichia coli* and Salmonella enterica isolates of human and animal origin in Lithuania. *Foodborne Pathogens and Disease*.

[42] Literak I, Dolejska M, Rybarikova J (2009). Highly variable patterns of antimicrobial resistance in commensal *Escherichia coli* isolates from pigs, sympatric rodents, and flies. *Microbial Drug Resistance*.

[43] Machado E, Coque TM, Cantón R, Sousa JC, Peixe L (2008). Antibiotic resistance integrons and extended-spectrum *β*-lactamases among Enterobacteriaceae isolates recovered from chickens and swine in Portugal. *Journal of Antimicrobial Chemotherapy*.

[44] Campbell LD, Scott HM, Bischoff KM, Anderson RC, Harvey RB (2005). Prevalence of class 1 integrons and antimicrobial resistance gene cassettes among enteric bacteria found in multisite group-level cohorts of humans and swine. *Journal of Food Protection*.

[45] Goldstein C, Lee MD, Sanchez S (2001). Incidence of class 1 and 2 integrases in clinical and commensal bacteria from livestock, companion animals, and exotics. *Antimicrobial Agents and Chemotherapy*.

[46] Abatih EN, Alban L, Ersbøll AK, Lo Fo Wong DM (2009). Impact of antimicrobial usage on the transmission dynamics of antimicrobial resistant bacteria among pigs. *Journal of Theoretical Biology*.

[47] McGowan JE (2001). Economic impact of antimicrobial resistance. *Emerging Infectious Diseases*.

[48] Krapac IG, Dey WS, Roy WR (2002). Impacts of swine manure pits on groundwater quality. *Environmental Pollution*.

[49] MacKie RI, Koike S, Krapac I, Chee-Sanford J, Maxwell S, Aminov RI (2006). Tetracycline residues and tetracycline resistance genes in groundwater impacted by swine production facilities. *Animal Biotechnology*.

[50] Byrne-Bailey KG, Gaze WH, Zhang L (2011). Integron prevalence and diversity in manured soil. *Applied and Environmental Microbiology*.

[51] Moura A, Henriques I, Ribeiro R, Correia A (2007). Prevalence and characterization of integrons from bacteria isolated from a slaughterhouse wastewater treatment plant. *Journal of Antimicrobial Chemotherapy*.

